# Midkine (MDK) in cancer and drug resistance: from inflammation to therapy

**DOI:** 10.1007/s12672-025-02941-1

**Published:** 2025-06-11

**Authors:** Berna Yıldırım, Kudret Kulak, Ayhan Bilir

**Affiliations:** 1 Department of Histology and Embryology, Faculty of Medicine, İstanbul Atlas University, İstanbul, Turkey; 2 Department of Pediatrics, Faculty of Medicine, İstanbul Atlas University, İstanbul, Turkey

**Keywords:** Midkine (MDK), Multidrug resistance, Inflammatory diseases, Cancer

## Abstract

Midkine (MDK) is a heparin-binding growth factor implicated in the pathogenesis of various diseases, including cancer, chronic inflammation, and multidrug resistance (MDR). While its expression is minimal in adult tissues, it becomes markedly elevated during embryogenesis and in response to injury, infection, or hypoxia. MDK modulates inflammatory responses by recruiting immune cells and enhancing proinflammatory cytokine production. In oncogenesis, it promotes tumor proliferation, angiogenesis, epithelial-to-mesenchymal transition (EMT), and therapeutic resistance. Elevated MDK levels are frequently associated with aggressive tumor behavior and poor clinical outcomes. This review synthesizes current knowledge on MDK’s expression profiles, molecular mechanisms, and functional roles across pathological conditions. It also discusses MDK’s emerging value as a diagnostic and prognostic biomarker, and highlights recent advances in therapeutic strategies including small molecule inhibitors, RNA-based approaches, and receptor-blocking peptides. Overall, MDK represents a promising target for future personalized therapies, although further preclinical and clinical validation is warranted to confirm its translational potential.

## Introduction

**Fig. 1:**
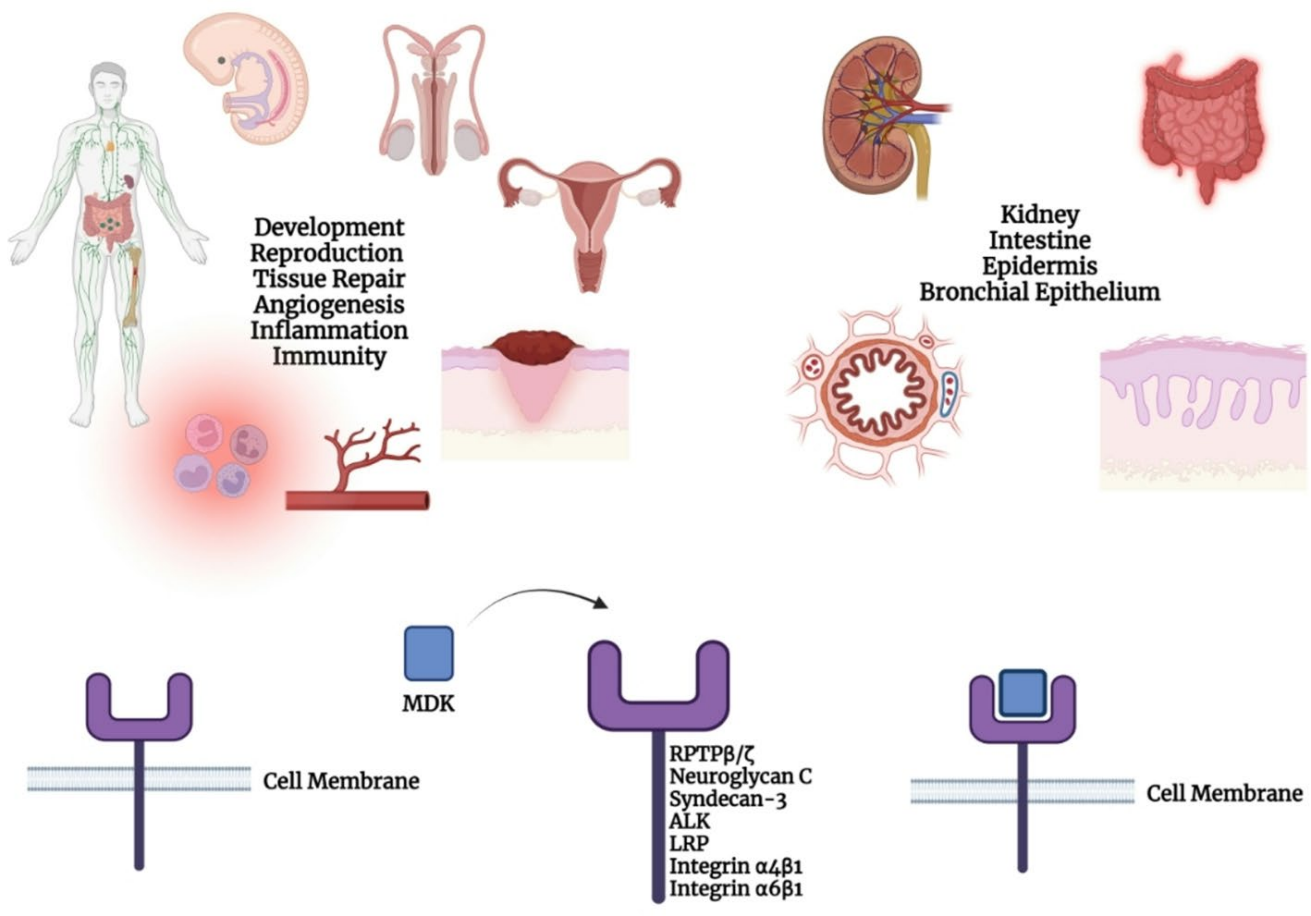
MDK receptor binding and associated physiological processes. Midkine (MDK) is a heparin-binding cytokine that exerts its biological functions through interactions with multiple cell surface receptors including anaplastic lymphoma kinase (ALK), receptor-type protein tyrosine phosphatase beta/zeta (RPTPβ/ζ), syndecan-3, neuroglycan C, low-density lipoprotein receptor-related protein (LRP), and integrins α4β1 and α6β1. These interactions trigger downstream signaling pathways involved in proliferation, differentiation, and survival. While MDK expression is low in adult tissues under normal conditions, it is markedly elevated in response to tissue injury, inflammation, or hypoxia. High MDK expression is observed in the kidney, intestine, epidermis, and bronchial epithelium. The illustration summarizes the receptor-mediated actions of MDK and highlights its pleiotropic roles in development, reproduction, tissue repair, angiogenesis, inflammation, and immune modulation. *(Created with BioRender.com)*

Heparin-binding cytokine Midkine (MDK) plays a crucial role in promoting cell motility, survival, and differentiation. The human MDK gene, located on chromosome 11 at p11.2, is flanked by the diacylglycerol kinase ζ gene and the muscarinic cholinergic receptor 4 gene [1, 2]. It contains four coding exons [3]. MDK is involved in several physiological processes, including development, reproduction, tissue repair, inflammation, immune responses, angiogenesis, and disease pathogenesis (Fig. 1) [4]. Its expression is markedly elevated during embryogenesis [4–6]. In adults, MDK is predominantly expressed in specific tissues such as the kidney, gastrointestinal tract, epidermis, and bronchial epithelium [4, 7–9]. Following tissue injury, MDK expression increases across various organs, particularly in macrophages and lymphocytes [10–13]. MDK is involved in embryogenesis and physiological processes in adult tissues through the receptors to which it binds, including ALK, LRP1, Notch2, and RPTPβ/ζ (Fig. 1) [14].

MDK exerts its biological functions by binding to a diverse range of receptors, including syndecan-3, anaplastic lymphoma kinase (ALK), and receptor protein tyrosine phosphatase (RPTP) β/ζ [15–19]. It also promotes neurite outgrowth in oligodendrocyte precursor-like cells through interactions with proteoglycans such as Neuroglycan C [14, 20, 21]. Additionally, MDK has been shown to bind to integrin α4β1, integrin α6β1, and low-density lipoprotein receptor-related protein (LRP) in the brain (Fig. 1) [13, 22, 23].

FGF-10 and Epidermal Growth Factor (EGF) are key regulators of MDK expression [24, 25]. MDK transcription is stimulated by ER-β activation, which subsequently activates protein kinase Cδ, while ER-α exerts a negative regulatory effect on MDK expression [13, 26, 27]. Additionally, retinoic acid has been shown to upregulate MDK levels [28]. Both MDK and pleiotrophin (PTN) expression are also induced under hypoxic conditions. In particular, hypoxia increases MDK expression in HUVECs and monocytes [29]. This response is mediated by direct transcriptional activation of the MDK promoter via hypoxia-inducible factor-1α (HIF-1α) [30].

The mechanisms through which MDK modulates cancer progression involve interactions with key receptors and downstream pathways [31]. Midkine (MDK) mediates its pleiotropic biological effects through interactions with several distinct cell surface receptors, including anaplastic lymphoma kinase (ALK), low-density lipoprotein receptor-related protein 1 (LRP1), and Notch2, among others [22, 32–34]. These interactions initiate intracellular signaling cascades critical for cancer progression, immune regulation, and tissue remodeling [35]. Binding of MDK to ALK has been shown to promote PI3K/AKT and MAPK pathway activation, enhancing cell survival and proliferation in neuroblastoma and glioma models, although the precise ligand-receptor relationship remains partially unresolved [36]. LRP1 acts as a high-affinity MDK receptor, facilitating endocytosis and nuclear translocation of MDK, and contributing to cell survival through activation of downstream effectors such as Akt and HIF-1α [32]. Furthermore, MDK is known to activate Notch2 signaling in cancer cells, leading to epithelial-mesenchymal transition (EMT), drug resistance, and aggressive tumor behavior [37]. In addition to these interactions, MDK influences Jak/STAT signaling, particularly STAT3, which has been implicated in inflammatory and oncogenic processes [38]. The convergence of these receptor-mediated pathways underscores MDK’s central role in modulating tumor microenvironment and therapeutic resistance, highlighting its potential as both a biomarker and a target for intervention [14].

## MDK in inflammation

**Fig. 2:**
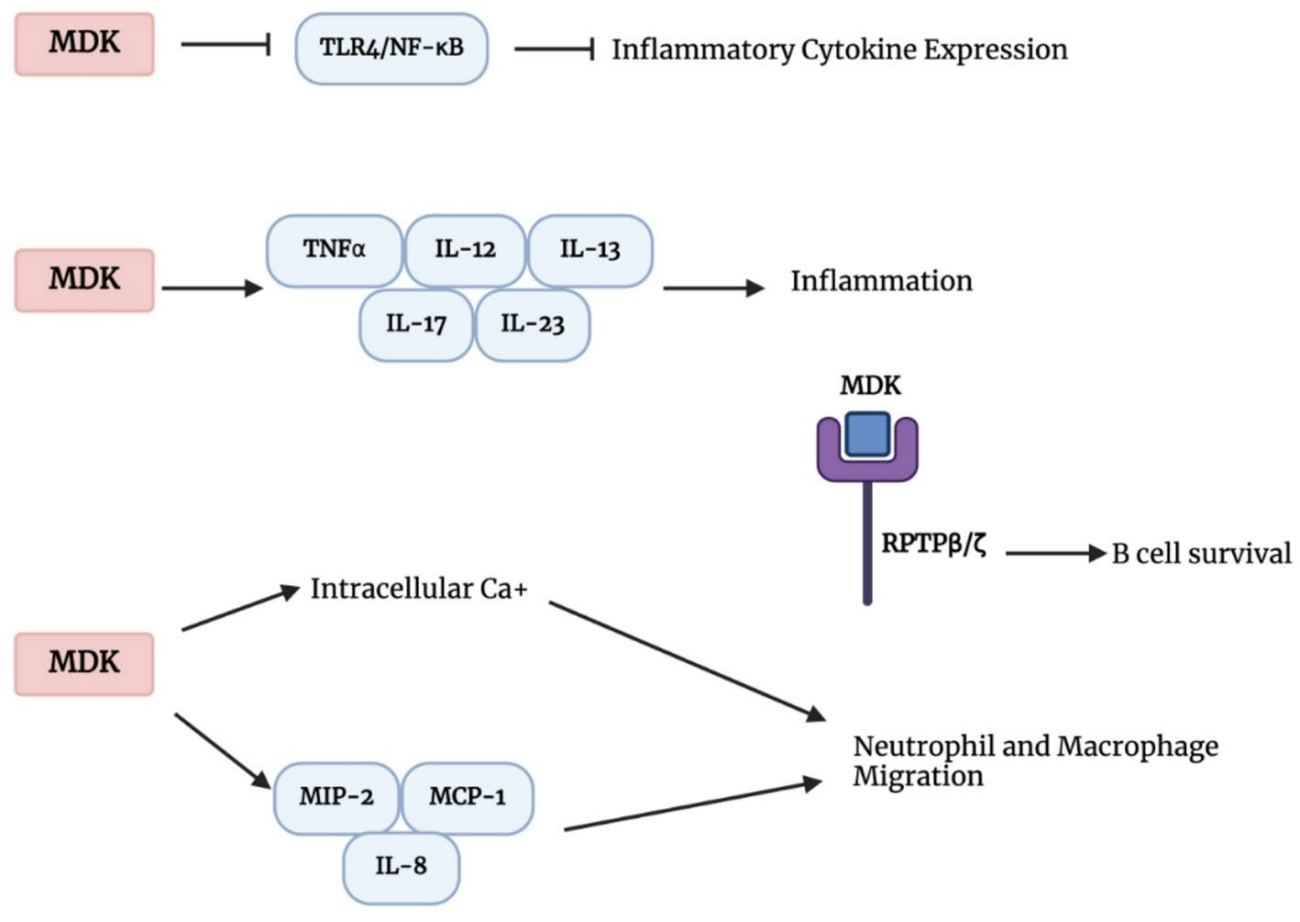
MDK’s role in inflammation and immune cell recruitment. Midkine (MDK) contributes to inflammation by activating Toll-like receptor 4 (TLR4) and NF-κB signaling pathways, resulting in the upregulation of inflammatory cytokines. MDK also promotes the expression of TNF-α, IL-12, IL-13, IL-17, and IL-23, which are critically involved in chronic inflammatory diseases such as Crohn’s disease and multiple sclerosis. In parallel, MDK enhances neutrophil and macrophage recruitment by upregulating chemokines including IL-8, MCP-1, and MIP-2, and by inducing intracellular calcium mobilization. Additionally, MDK binding to RPTPβ/ζ has been shown to promote B cell survival, contributing to immune regulation. This figure illustrates the molecular interactions through which MDK orchestrates both innate and adaptive immune responses in inflammatory microenvironments. *(Created with BioRender.com)*

MDK may enhance inflammatory responses by promoting the migration of leukocytes such as neutrophils and macrophages, stimulating chemokine production, and inhibiting the generation of regulatory T cells [13, 21, 39–42]. Through its interaction with RPTP-β, MDK supports B cell survival both in vitro and in vivo [10]. A significant portion of MDK binding occurs at the chondroitin sulfate domain of RPTPβ/ζ, which is essential for its neuroprotective effects [18]. Notably, MDK has no observable effect on B cells isolated from RPTP-β-null mice [10]. Interestingly, these RPTP-β-deficient B cells also fail to respond to Hepatocyte Growth Factor (HGF) and macrophage Migration Inhibitory Factor (MIF) [10]. This observation suggests that increased MDK expression could potentially enhance B cell survival [10]. Moreover, RPTP-β-null mice exhibit reduced numbers of mature B cells in the spleen, lymph nodes, and bone marrow, supporting a role for MDK/RPTP-β signaling in the maintenance of B cell populations within lymphoid tissues [13]. By increasing the migration of inflammatory leukocytes such neutrophils and macrophages, increasing chemokine synthesis, and inhibiting the generation of regulatory T cells, MDK may enhance the inflammatory response [13, 21, 39–42]. MDK plays a role in processes such as regulation of inflammatory response, neutrophil and macrophage migration, and TLR4/NF-κB signaling activation in trauma and sepsis models (Fig. 2) [43].

It has been shown that elevated cytokine and chemokine release can suppress neutrophil migration [44]. Acting as both a chemotactic and haptotactic agent for neutrophils and macrophages, MDK may play a role in this regulatory process [45, 46]. In vivo models deficient in MDK display significantly reduced leukocyte migration [41]. MDK influences neutrophil function both directly and indirectly [45]. Its direct effects include induction of intracellular calcium mobilization [45]. Indirectly, MDK upregulates the expression of IL-8, Monocyte Chemotactic Protein-1 (MCP-1), and Macrophage Inflammatory Protein-2 (MIP-2), enhancing the inflammatory response (Fig. 2) [42, 47]. Additionally, MDK can regulate excessive nitric oxide (NO) release that inhibits neutrophil migration by suppressing Bradykinin-induced NO production [48, 49]. Elevated MDK levels have been implicated in impaired neutrophil chemotaxis observed in sepsis [45].

MDK is highly expressed in traumatic brain injury (TBI); however, its downregulation reduces macrophage and microglial infiltration into the injured tissue and alters their polarization state [50]. This modulation leads to decreased neuroinflammation and improved neurological outcomes following TBI [50]. One study reported a significant increase in MDK levels in the cerebral cortex of TBI-induced rats. In vitro suppression of MDK reduced the production of inflammatory cytokines, inhibited the TLR4/NF-κB signaling pathway in microglia, and attenuated microglia-mediated astrocyte activation [51]. These results suggest that MDK acts as a proinflammatory mediator by activating the TLR4/NF-κB pathway in both microglia and astrocytes (Fig. 2) [51]. Additionally, HOXA11-AS was shown to upregulate MDK expression, whereas overexpression of miR-124-3p suppressed it [51]. In a separate study, MDK-knockout (MDK−/−) animals exhibited decreased activation of microglia and macrophages in the perilesional area and reduced expression of M1 markers such as TNF-α and CD11b, indicating that MDK may regulate microglia/macrophage polarization and inflammatory responses after TBI [50]. Furthermore, during the acute phase post-TBI, an increase in cells expressing M2 phenotype markers (CD163, arginase-1) was observed [50]. Arginase-1 is considered essential for central nervous system repair, while TNF-α is a key glia-derived cytokine involved in caspase-3-dependent neuronal apoptosis [52, 53]. Collectively, these findings suggest that MDK deficiency may mitigate neuroinflammation and neuronal cell death following TBI by modulating M1/M2 polarization of microglia and macrophages [50].

A study reported that patients recently diagnosed with Multiple Sclerosis (MS) and Neuromyelitis Optica exhibited higher MDK levels compared to healthy individuals [54]. Elevated MDK expression is believed to contribute to the release of inflammatory cytokines, suppression of neuronal cell growth, and potentially to disease progression [55]. In the experimental autoimmune encephalomyelitis (EAE) model—an established animal model of MS—MDK expression increases during disease induction [54]. EAE pathogenesis has been strongly linked to Th17 cells, a subset of CD4 + T lymphocytes [56–58]. Previous studies have shown that CD4 + T cells express MDK during EAE progression, with Th1 cells displaying significantly higher MDK expression in vitro compared to naïve Th cells [59]. These findings suggest that MDK may act as a contributing factor in Th1 cell-mediated EAE [59]. Suppressing MDK activity could potentially prevent the development of EAE [59]. Supporting this, an anti-MDK aptamer has demonstrated suppressive effects in experimental models [40]. Moreover, MDK-deficient animals exhibit reduced Th1 and Th17 activity, along with significantly lower levels of their associated cytokines, indicating that MDK may serve as a critical regulator of autoimmune responses [21].

In both inflammatory and malignant conditions—particularly in inflammatory bowel diseases such as Crohn’s disease and ulcerative colitis, as well as in MS and rheumatoid arthritis (RA)—MDK has emerged as a cytokine of diagnostic significance [60]. A variety of cytokines have been implicated in intestinal inflammation [61]. Elevated levels of TNF-α, IL-1β, and IL-6 have been linked to disease activity and inflammation in Crohn’s disease (CD) patients [54, 62]. Additional studies have identified IL-12, IL-13, IL-17, and IL-23 as critical cytokines in CD pathogenesis [63–66]. This proinflammatory immune response, largely driven by Th1 cells, is characterized by high expression of TNF-α, IL-1β, IL-12, and IL-13 [61]. High MDK levels have been associated with these cytokines [61]. These findings suggest that elevated MDK concentrations in CD patients correlate with increased inflammatory activity and clinical disease severity [61]. Therefore, MDK functions as an endogenous proinflammatory mediator that may contribute to intestinal inflammation through TNF-α, IL-1β, IL-12, IL-13, IL-17, and IL-23 signaling (Fig. 2) [61]. Due to its close association with disease activity, MDK is considered an important biomarker in both Crohn’s disease and celiac disease (CeD) [60].

MDK has been shown to be upregulated in response to ischemic tissue damage in organs such as the kidney, heart, brain, and blood vessels [13, 21, 41, 42]. By promoting the survival of injured cells and exhibiting antimicrobial properties, MDK may contribute to tissue repair and regeneration [67–71]. Elevated levels of proinflammatory cytokines have been associated with increased MDK expression [72]. In a previous study, TNF-α and IL-1β were shown to induce MDK expression in 3Y1 cells in a time-dependent manner [72]. In the IEC-6 intestinal epithelial wound healing model, several growth factors and cytokines have been reported to regulate epithelial repair via TGF-β signaling [73, 74]. MDK enhances IEC-6 cell migration without affecting proliferation, a pattern commonly observed in TGF-β-mediated repair processes [74, 75]. These findings suggest that MDK may promote wound healing by inducing TGF-β and stimulating epithelial cell migration in the IEC-6 monolayer model [72]. In addition, in an ischemic kidney injury model, increased MDK expression has been linked to elevated levels of chemokines such as MIP-2 and MCP-1 [42]. MDK deficiency significantly reduces the expression of these chemokines and attenuates tubulointerstitial injury [42]. Moreover, MDK-deficient animals exhibit decreased infiltration of neutrophils and monocytes/macrophages into the kidney in ischemic injury and into the arterial wall in restenosis models [41].

Toll-Like Receptors (TLRs), the upregulation of inflammation-associated molecules, and activation of innate immunity have all been implicated in neuroinflammatory processes [23]. The expression levels of MDK in both developing and mature nervous systems have been extensively studied across species, highlighting its importance in neuron–glia interactions [76–78]. Following injury, MDK expression markedly increases in various cell types, including inflammatory macrophages and microglia [21, 23, 79–81]. These cells contribute to processes such as wound healing and neuronal survival [82, 83]. Research has shown that MDK is frequently expressed in ischemic regions in rats with acute cerebral infarction [84]. Beyond ischemic injury, MDK accumulation has been observed in the substantia nigra of Parkinson’s disease (PD) patients and within senile plaques and serum of individuals with Alzheimer’s disease (AD) [83]. Additionally, MDK is present in glial cytoplasmic inclusions in brains affected by multiple system atrophy [85]. Elevated MDK expression has also been noted in response to neuroinflammatory conditions and after exposure to substances of abuse such as amphetamines, alcohol, and opioids, as well as following various types of brain trauma including ischemia [83].

## Multidrug resistance in bacterial and viral diseases

Multidrug resistance (MDR) refers to the ability of microorganisms and cancer cells to resist the effects of various chemotherapeutic agents [86]. This resistance is often driven by the overexpression of proteins that actively expel chemotherapy drugs, thereby reducing their intracellular concentrations to subtherapeutic levels [87]. MDR is responsible for tens of thousands of cancer-related deaths annually and is commonly mediated by cellular transport systems, particularly Adenosine Triphosphate Binding Cassette (ABC) transporters [88]. These pumps facilitate the efflux of a wide range of substrates, including amino acids, peptides, ions, carbohydrates, toxins, lipids, and pharmaceutical compounds [89]. MDR significantly affects the treatment outcomes of multiple serious human diseases, including cancer, infections, and immune system disorders [89].

In bacteria, MDR arises through mechanisms such as the upregulation of resistance-conferring genes, overproduction of efflux pumps, or the enzymatic inactivation of drugs [90]. For instance, antibiotics like penicillin and tetracycline may become ineffective due to hydrolysis or chemical modification [91]. Horizontal gene transfer (HGT) is a key process that enables the rapid acquisition and spread of resistance traits across bacterial populations [92]. Additionally, bacterial biofilm formation represents another critical factor contributing to antibiotic resistance by providing a protective environment for microbial survival [93].

Although classical bacterial multidrug resistance primarily involves mechanisms such as drug inactivation, efflux pumps, and horizontal gene transfer, recent findings suggest that host-derived molecules like MDK may indirectly influence bacterial persistence and immune evasion [94, 95]. MDK has been shown to possess antimicrobial properties, particularly through modulation of immune cell recruitment and cytokine responses [96–98]. Its high expression in barrier tissues such as the airway and gastrointestinal epithelium supports its role in mucosal defense [99]. Thus, while MDK does not directly confer antibiotic resistance, its role in shaping the immune microenvironment may be relevant in chronic infections and biofilm-associated bacterial survival [100].

The widespread misuse and overuse of antibiotics, combined with their limited effectiveness against biofilm-associated infections (BRIs), have led to a substantial rise in multidrug-resistant (MDR) bacterial strains [86]. MDR poses serious health risks, particularly in infections caused by carbapenem-resistant Enterobacterales and *Klebsiella pneumoniae* [101]. Multidrug-resistant tuberculosis (MDR-TB) remains a major global health threat, often requiring prolonged, toxic, and costly treatment regimens [102]. Resistance is also increasing in fungal pathogens such as *Candida auris* and *Aspergillus fumigatus* [99]. Promising alternatives, including phage therapy and other innovative strategies, are currently under investigation to improve treatment outcomes against MDR microorganisms [100]. Nonetheless, further research and novel therapeutic approaches are urgently needed to address this growing public health challenge [13, 103–118].

MDR in viruses also presents a critical concern, particularly in infections caused by Human Immunodeficiency Virus (HIV), Hepatitis C Virus (HCV), and influenza [119]. Antiviral resistance typically arises from mutations in viral target proteins, which disrupt the binding or function of therapeutic agents [120]. For example, strains of H1N1 influenza A virus have been identified that are resistant to oseltamivir and similar antiviral drugs [121–124]. Similarly, the emergence of MDR HIV-1 strains has limited available treatment options [125]. During the COVID-19 pandemic, the widespread and often empirical use of antibiotics contributed to the acceleration of MDR bacterial spread, particularly among Gram-negative organisms [126–128]. However, further studies are needed to fully understand the long-term impact of the pandemic on global antimicrobial resistance trends [129, 130].

Recent findings implicate MDK in the immunopathology of viral infections, including its upregulation during SARS-CoV-2 infection and its contribution to neutrophil infiltration and lung fibrosis via NOX1 and NOTCH2 signaling [131, 132]. Although MDK is not directly implicated in classical viral drug resistance mechanisms such as reverse transcriptase mutations or protease inhibitor escape, its involvement in immune dysregulation may indirectly contribute to disease severity and therapeutic inefficacy [133]. Therefore, while the concept of MDR in viral diseases primarily revolves around genetic resistance, MDK’s immunomodulatory role deserves further exploration, particularly in inflammation-driven pathologies such as COVID-19 [134].

**Fig. 3:**
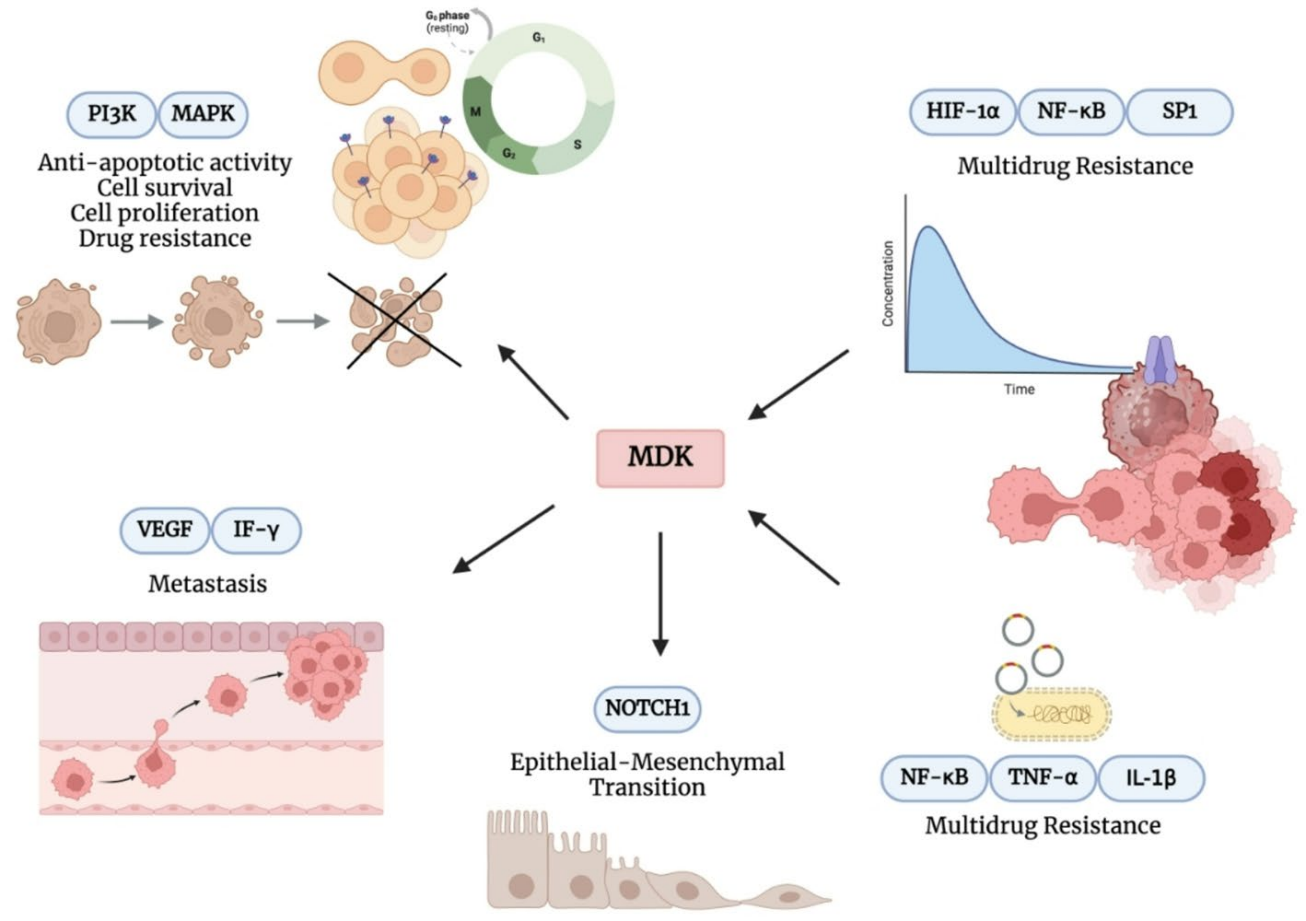
MDK’s role in epithelial-mesenchymal transition,multidrug resistance, and tumor progression. Midkine (MDK) contributes to cancer progression through multiple interconnected mechanisms. By activating signaling pathways such as PI3K/AKT and MAPK, MDK promotes cell survival, proliferation, and resistance to apoptosis. It also plays a central role in multidrug resistance (MDR) by upregulating drug efflux transporters and modulating transcriptional regulators including HIF-1α, NF-κB, and SP1. MDK enhances epithelial-mesenchymal transition (EMT) via the NOTCH1/2 signaling axis, contributing to increased cellular motility and metastatic potential. In addition, MDK is associated with increased angiogenesis through VEGF induction, and facilitates metastasis by promoting immune evasion and extracellular matrix remodeling. The cumulative effect of these processes results in more aggressive tumor phenotypes and resistance to conventional chemotherapies. *(Created with BioRender.com)*

MDK shares several key characteristics with antimicrobial proteins of the innate immune system, including heparin-binding domains, immune cell recruitment, and activation capabilities [94]. It has been recognized for its notable bactericidal and fungicidal properties [94]. High concentrations of MDK are found in barrier tissues such as the skin and major airways [94]. Its expression is regulated by molecules like retinoic acid (RA), which plays an essential role in immune function and organogenesis [95]. RA synthesis is promoted by TLR2, IL-4, and GM-CSF, while it is suppressed by prostaglandin E2 (PGE2) [94]. Inflammatory conditions that alter RA levels may influence MDK transcription [97, 98]. Nuclear factor kappa B (NF-κB) is a central regulator of MDK expression [96]. As a transcription factor involved in immune gene activation, NF-κB induces MDK through direct interaction with its promoter [135]. Its activation is triggered by various stimuli, including reactive oxygen species (ROS), TNF-α, IL-1β, and lipopolysaccharide (LPS) derived from Gram-negative bacteria (Fig. 3) [136].

Multiple studies have reported interactions between MDK and cytokines or chemokines associated with SARS-CoV-2 infection [133, 134, 137–139]. In COVID-19, TNF-α has been shown to both induce and enhance MDK expression [140]. MDK contributes to neutrophil infiltration and promotes lung remodeling and fibrosis via collagen deposition through NOX1-, MDK-, and NOTCH2-dependent signaling pathways [131]. These findings suggest that MDK may play a role in the pathogenesis of pulmonary fibrosis, a major complication of SARS-CoV-2 infection [131]. MDK is proposed to influence these outcomes by modulating inflammatory cell migration and the synthesis of various inflammatory mediators [132, 141].

## Multidrug resistance in Cancer

Recent studies suggest a strong association between MDK expression, multidrug resistance (MDR), and cancer development [142–147]. MDK contributes to MDR through multiple mechanisms, including the induction of drug efflux pumps, promotion of epithelial-mesenchymal transition (EMT), and stimulation of pro-proliferative molecule expression [13, 144, 148–151]. Several transcriptional regulators—such as HIF-1α, NF-κB, and SP1—have been shown to tightly control MDK expression [30, 152, 153]. These regulatory pathways may enhance the ability of cancer cells to acquire resistance through MDK overexpression (Fig. 3) [148, 154].

Resistance to apoptosis is a hallmark of cancer [155]. Mutations that impair apoptotic mechanisms contribute to tumor development and progression [155]. Cancer therapies such as chemotherapy and radiotherapy often aim to induce mitochondria-dependent apoptosis in malignant cells [156]. However, alterations in apoptotic signaling are associated with treatment resistance [156]. MDK has been shown to activate the PI3K and MAPK pathways, promoting cell survival, proliferation, and anti-apoptotic activity in tumor cells [157]. Notably, MDK is overexpressed in hepatocellular carcinoma (HCC), where it contributes to both drug resistance and apoptosis inhibition [158]. Targeting the IGF-1 receptor (IGF-1R) via RNA interference (RNAi) has been shown to suppress MDK expression, thereby reducing HCC growth and invasiveness [159]. Low-density lipoprotein receptor-related protein 1 (LRP1), a known MDK receptor, initiates oncogenic signaling upon binding MDK [160]. MDK-TRAP, a peptide derived from LRP1, binds MDK with high affinity and competitively blocks its interaction with LRP1, potentially preventing downstream oncogenic effects [160, 161]. Through these mechanisms, MDK plays a pivotal role in tumor initiation and progression, making it a compelling molecular target for cancer therapy [162].

The pro-angiogenic role of MDK is closely linked to its capacity to stimulate endothelial cell proliferation [163]. However, findings regarding its interaction with major angiogenic factors—such as VEGF-A—remain somewhat inconsistent [29, 163–165]. Some studies propose that elevated MDK expression may independently promote neovascularization, functioning as an alternative angiogenic mechanism [166]. It has been hypothesized that MDK drives tumor growth via an autocrine mechanism, while concurrently promoting endothelial cell proliferation through paracrine signaling—an idea supported by the observation that MDK is primarily expressed in tumor cells [166]. By enhancing the production of VEGF and other angiogenic mediators, MDK may worsen prognosis and facilitate tumor progression [166]. In cancers such as oral squamous cell carcinoma (OSCC), MDK has been identified as a potential target for anti-angiogenic therapy [166].

MDK has also been implicated in the regulation of epithelial-mesenchymal transition (EMT). Studies show that estradiol (E2) induces EMT via MDK, facilitating a mesenchymal phenotype enriched with drug efflux pumps and anti-apoptotic proteins [13, 162, 167]. In A549 human alveolar epithelial cells, hypoxia-induced activation of HIF-1α through a PKC-δ-dependent pathway was shown to upregulate MDK expression and subsequently promote EMT [26, 38]. Furthermore, MDK activates the NOTCH1 signaling pathway, which is known to induce EMT [144, 162]. Interestingly, another study reported that binding of MDK to NOTCH2 activated angiotensin-converting enzyme, resulting in elevated levels of angiotensin II—a key factor in the mechanical stretching of pulmonary epithelial cells during EMT [38, 168]. High MDK expression has also been associated with enhanced drug efflux capacity in childhood acute lymphoblastic leukemia (ALL), suggesting a role in multidrug resistance (MDR) [148]. In addition, increased MDK expression was observed in all drug-resistant cancer cell lines in a separate study, further supporting its involvement in MDR mechanisms across human malignancies [169].

Several studies suggest that MDK gene expression may protect cancer cells against various chemotherapeutic agents, including doxorubicin, cannabinoids, and adriamycin [146, 169–171]. Elevated MDK expression has also been associated with cisplatin resistance in renal and oral squamous cell carcinoma cells [172]. In primary lung cancer cells, an inverse correlation between MDK expression and cisplatin cytotoxicity has been reported [173, 174]. These findings imply that MDK contributes to chemotherapy resistance by attenuating the cytotoxic effects of cisplatin [175]. Bioinformatic analyses have identified MDK as one of the key genes implicated in the progression, metastasis, and drug resistance of ovarian cancer [176]. n one study, transfection of the MDK gene into HeLa cells—originally lacking MDK expression—conferred resistance to 5-fluorouracil (5-FU) and doxorubicin, and partial resistance to cisplatin. Conversely, siRNA-mediated silencing of MDK in SNU-638 human gastric cancer cells led to increased sensitivity to these agents, including 5-FU, doxorubicin, and cisplatin [177].

A study reported that MDK initiates cell-protective signaling against doxorubicin by activating the AKT signaling pathway [178]. In contrast, another study demonstrated that MDK can suppress cisplatin resistance in certain ovarian cancer cell lines [175]. Elevated MDK levels in stromal cells and cancer-associated fibroblasts (CAFs) have been shown to promote cisplatin resistance, while MDK was also found to reduce cisplatin cytotoxicity in AGS human gastric cancer cells, partly through alterations in the Notch signaling pathway [176]. Additionally, MDK has been shown to exert a protective effect against cisplatin-induced damage by upregulating Bcl-2 expression in various tumors [142]. In lymphoblastic leukemia cells, MDK overexpression enhanced the efflux of chemotherapeutic agents, contributing to multidrug resistance [179]. Collectively, these findings highlight MDK’s crucial role in tumor progression and support its potential as a therapeutic target [148].

Interferons (IFs) are a group of cytokines with antiviral, antiproliferative, and immunomodulatory functions [162]. Among them, interferon-gamma (IF-γ) is widely used in cancer therapy; however, recent studies suggest that it may also facilitate metastasis in certain contexts [162]. Research indicates that MDK functions as an IF-γ response element, mediating IF-γ-induced metastasis across multiple cancer types [180]. In malignant melanoma, MDK secreted by tumor cells promotes immune suppression and facilitates immune evasion, thereby enhancing tumor development [181]. These findings further support the consideration of MDK as a candidate for gene therapy and underscore its involvement in drug resistance mechanisms [169].

## Therapeutic potential of the MDK

Neutrophils are the principal innate immune cells involved in regulating the body’s inflammatory response [182]. However, prolonged neutrophil infiltration has been associated with the onset and progression of various human diseases, potentially resulting in long-term tissue damage [182, 183]. A study demonstrated that MDK stimulates the production of inflammatory cytokines and induces NETosis (neutrophil extracellular trap formation) both in vitro and in vivo [183]. These findings suggest that MDK may serve as a promising molecular target for anti-inflammatory therapies [60, 184].

MDK inhibitors have shown therapeutic efficacy in several diseases, including renal disorders, postoperative hypertension, rheumatoid arthritis (RA), multiple sclerosis (MS), and various cancers [13]. MDK also contributes to pathological angiogenesis [13, 60]. In a diabetic kidney disease (DKD) model, antisense oligonucleotides (ODNs) targeting MDK significantly improved renal function and reduced tissue damage by suppressing MDK expression [183]. Furthermore, the inhibition or downregulation of MDK using RNA interference (RNAi) or siRNA has been shown to enhance the effectiveness of chemotherapeutic agents by restoring drug sensitivity in resistant cancer cells [148, 162].

Overexpression of MDK is associated with numerous malignancies and represents a potential target for personalized therapy, as well as a biomarker for diagnosis and prognosis [31, 162, 185]. As a secreted cytokine, MDK is detectable in biological fluids such as blood, urine, cerebrospinal fluid, or tumor-derived mRNA, making it a cost-effective tool for early diagnosis and prognostic evaluation [186, 187]. An RNA aptamer targeting MDK has demonstrated therapeutic benefits without significant side effects, increasing its potential for clinical application [188]. Further research into MDK’s mechanisms of action, chronic toxicity, and optimal dosing regimens will be essential for translating these findings into therapy [188]. Serum MDK levels may also serve as effective tumor markers. Both the MDK protein and its promoter region are under investigation as molecular targets for gene therapy [185, 189]. Ongoing translational research aims to establish MDK as a viable target in gene therapy and cancer treatment, offering a novel approach in oncology [169]. Future studies evaluating MDK blood levels and their correlation with prognosis may further validate its utility as a tumor biomarker [169]. These therapeutic approaches are summarized in Table 1, which presents the main MDK-targeted strategies, their mechanisms of action, and developmental stages. Table 2 provides an overview of MDK expression levels in various cancers and highlights its relevance as a diagnostic and prognostic biomarker. Several therapeutic approaches have been developed to target MDK, including small molecule inhibitors, RNA-based strategies, and receptor-binding peptides. These strategies are at various stages of preclinical development and have shown promising results in vitro and in vivo.

**Table 1:** MDK-Targeted therapeutic strategies

Strategy Type	Example Molecule/Tool	Mechanism of Action	Clinical Stage / Data Status	References
Small molecule inhibitor	iMDK	Inhibits MDK signaling, suppresses tumor growth	Preclinical (in vitro and in vivo)	[151, 162]
RNA interference (RNAi)	siRNA targeting MDK	Suppresses MDK gene expression	Preclinical	[31, 151, 162]
RNA aptamer	MDK-specific RNA aptamer	Binds to MDK and neutralizes its activity	Preclinical (mouse models, low toxicity)	[188]
Antisense oligonucleotide (ODN)	Anti-MDK ODN	Reduces MDK mRNA stability	Preclinical (kidney disease model)	[183]
Receptor-binding peptide (MDK-TRAP)	LRP1-derived MDK-TRAP	Blocks MDK-LRP1 interaction and downstream signaling	Experimental (binding studies)	[160, 161]

**Table 2:** MDK expression and biomarker role in various cancers

Cancer Type	MDK Expression Level	Clinical Relevance	Comparison with Other Biomarkers	References
Hepatocellular carcinoma (HCC)	High	Associated with poor prognosis and drug resistance	Earlier than AFP	[158, 190]
Lung cancer	Elevated	Correlates with cisplatin resistance	More specific than CEA	[172–174]
Ovarian cancer	Upregulated	Linked to tumor progression and chemoresistance	Complementary to CA125	[168]
Prostate cancer	Moderate	Potential early diagnostic marker	Used with PSA	[186, 187]
Melanoma	Increased	Supports immune escape and tumor growth	Potential alternative to S100B	[181]

## Discussion

The critical role of MDK in immune system modulation underscores its impact on inflammatory diseases. By facilitating the migration of immune cells such as neutrophils and macrophages into affected tissues, MDK contributes to inflammation-induced tissue damage observed in conditions like multiple sclerosis, inflammatory bowel disease, and traumatic brain injury. This effect is compounded by MDK’s activation of Toll-like receptors and NF-κB signaling, which enhances the release of proinflammatory cytokines. Given that chronic inflammation is a known driver of tumorigenesis, MDK’s dual involvement in inflammatory and malignant processes reinforces its value as a therapeutic target.

MDK also plays a key role in tumor progression by promoting angiogenesis and epithelial-to-mesenchymal transition (EMT), mechanisms that enhance tumor invasiveness and metastasis. Furthermore, its contribution to multidrug resistance (MDR)—through the upregulation of drug efflux pumps—represents a major obstacle in chemotherapy, leading to reduced drug efficacy and therapeutic failure. These features make MDK a compelling molecular target in oncology. This is further supported by recent omics-based investigations that have identified MDK as a critical molecular player in tumor aggressiveness and drug resistance. Transcriptomic and proteomic analyses from large-scale datasets such as TCGA and CPTAC have demonstrated that MDK is consistently overexpressed in aggressive tumor types, including hepatocellular carcinoma, lung adenocarcinoma, and ovarian cancer. These studies also revealed correlations between MDK overexpression and elevated levels of anti-apoptotic markers (e.g., BCL2, MCL1), EMT-related genes, and drug resistance transporters such as ABCG2 and MDR1. Moreover, single-cell RNA sequencing in glioblastoma and melanoma has highlighted MDK as a niche-specific stromal factor contributing to immune evasion and resistance to checkpoint blockade therapies. Proteomics-based pathway mapping further suggests that MDK modulates integrin and NOTCH signaling pathways, supporting its role in metastatic potential and chemoresistance (as supported by transcriptomic and proteomic studies [151, 191, 192]). The integration of these omics data provides robust molecular validation of MDK’s involvement in tumor progression and underscores its relevance as a candidate for precision oncology.

Midkine (MDK) drives tumor aggressiveness and therapeutic resistance by engaging multiple molecular mechanisms. It activates the PI3K/AKT and MAPK signaling cascades primarily through interaction with ALK receptors, thereby enhancing cell proliferation, survival, and anti-apoptotic signaling, particularly in neuroblastoma and glioma models. In hypoxic tumor microenvironments, MDK expression is induced via HIF-1α, which further contributes to epithelial-to-mesenchymal transition (EMT) and metastatic behavior. Additionally, MDK triggers NOTCH1 and NOTCH2 signaling, pathways known to promote EMT and chemoresistance in multiple cancer types. Its downstream targets include anti-apoptotic proteins such as Bcl-2, and MDK also facilitates drug efflux by upregulating ABC transporters, such as MDR1 and ABCG2, leading to multidrug resistance in gastric, leukemia, and ovarian cancer models. These mechanistic insights underscore MDK’s critical role in orchestrating drug resistance and metastatic progression, and provide a rationale for targeting MDK in combination therapies.

Therapeutic strategies aimed at silencing MDK expression—such as RNA aptamers and siRNA—have shown promise in preclinical models, with potential benefits in both cancer and chronic inflammatory diseases. In addition, MDK is emerging as a valuable prognostic and predictive biomarker, particularly in malignancies characterized by MDR. However, the long-term safety and effectiveness of MDK-targeted therapies remain to be fully evaluated. Potential adverse effects, such as immunosuppression or unintended disruption of physiological functions, must be carefully assessed. Future studies should aim to optimize MDK inhibition strategies, define the most responsive patient populations, and incorporate these approaches into personalized treatment frameworks.

Several therapeutic strategies have been developed to inhibit MDK activity, including small molecule inhibitors, RNA-based technologies, and receptor-binding peptides. The small molecule inhibitor iMDK has demonstrated strong tumor suppressive activity in preclinical models by directly blocking MDK-mediated signaling and inducing apoptosis. RNA interference (RNAi) and siRNA approaches effectively downregulate MDK expression, restoring chemosensitivity in resistant cancer cell lines such as gastric, lung, and leukemic models. RNA aptamers targeting MDK have shown therapeutic efficacy in mouse models with minimal toxicity, offering translational potential. Antisense oligonucleotides (ODNs) have also been explored, particularly in kidney disease models, showing reduced tissue damage and improved function. In addition, a receptor-binding peptide known as MDK-TRAP has been designed to disrupt the MDK-LRP1 interaction and downstream oncogenic signaling. Although these approaches are largely in preclinical stages, they highlight the growing therapeutic interest in MDK inhibition. However, challenges remain, such as targeted delivery, stability, and off-target effects, which must be addressed in future studies to ensure clinical success.

## Conclusion

The diverse roles of MDK in immune modulation, angiogenesis, and multidrug resistance make it a key factor in the pathogenesis of both inflammatory diseases and cancer. A deeper understanding of its molecular functions—particularly its regulation of immune responses and drug resistance mechanisms—may enable the development of novel therapeutic strategies. Targeting MDK holds substantial promise for advancing personalized and more effective treatment approaches. In addition, MDK is emerging as a valuable biomarker for early diagnosis and prognosis, offering insights into disease progression and therapeutic response. Nevertheless, further research is necessary to validate its clinical utility and to optimize MDK-targeted interventions for safe and effective application.

## Data Availability

No datasets were generated or analysed during the current study.
